# Machine learning for privacy‐protected voice analysis in dementia assessment

**DOI:** 10.1002/alz.091272

**Published:** 2025-01-09

**Authors:** Meysam Ahangaran, Nauman Dawalatabad, Cody Karjadi, James Glass, Rhoda Au, Vijaya B. Kolachalama

**Affiliations:** ^1^ Boston University Chobanian & Avedisian School of Medicine, Boston, MA USA; ^2^ Massachusetts Institute of Technology, Cambridge, MA USA; ^3^ The Framingham Heart Study, Framingham, MA USA

## Abstract

**Background:**

The prevalence of cognitive impairments, such as mild cognitive impairment (MCI) and Alzheimer's disease (AD), has surged, necessitating rapid, cost‐effective, and non‐invasive diagnostic tools. Speech, as a rich source of cognitive indices, offers a promising avenue for distinguishing between healthy controls, MCI, and AD groups. However, the utilization of voice data poses privacy challenges, as speaker identities can be discerned through automatic speaker verification systems.

**Method:**

We developed a machine learning framework for dementia assessment, utilizing acoustic features obtained through advanced speaker anonymization techniques. Our work leveraged a wealth of digital voice recordings from the Framingham Heart Study, which, since 2005, has captured extensive spoken responses to neuropsychological tests. These recordings were instrumental in refining our privacy‐conscious algorithmic approach to dementia assessment.

**Result:**

The developed tool demonstrated efficacy in de‐identifying speakers while maintaining the integrity of cognitive features in speech. This retention allows for the continued development of vocal biomarkers that correlate with neuropsychological assessments and cognitive impairment diagnoses.

**Conclusion:**

Our approach unites the need for privacy with the utility of voice data in dementia assessment. It offers a scalable, accessible solution that could revolutionize early cognitive decline detection, while also aligning with the increasing emphasis on patient privacy in medical research.

